# LGBTQIA+ inclusion in the global health policy agenda: A critical discourse analysis of the Lancet Commission report archive

**DOI:** 10.1371/journal.pone.0311506

**Published:** 2024-10-04

**Authors:** William E. Rosa, Sofia Weiss Goitiandia, Debbie Braybrook, Nicholas Metheny, Kailey E. Roberts, Meghan McDarby, Mia Behrens, Cathy Berkman, Gary L. Stein, Adebola Adedimeji, Donna Wakefield, Richard Harding, Dingle Spence, Katherine Bristowe

**Affiliations:** 1 Department of Psychiatry and Behavioral Sciences, Memorial Sloan Kettering Cancer Center, New York, NY, United States of America; 2 Florence Nightingale Faculty of Nursing Midwifery and Palliative Care, Cicely Saunders Institute, King’s College London, London, England, United Kingdom; 3 School of Nursing and Health Studies University of Miami, Coral Gables, FL, United States of America; 4 Ferkauf Graduate School of Psychology, Yeshiva University, New York, NY, United States of America; 5 Graduate School of Social Service, Fordham University, New York, NY, United States of America; 6 Wurzweiler School of Social Work, Yeshiva University, New York, NY, United States of America; 7 Department of Social Sciences and Health Policy, School of Medicine, Wake Forest University, Winston-Salem, NC, United States of America; 8 Faculty of Medical Sciences, Population Health Sciences Institute, Newcastle University, Newcastle upon Tyne, United Kingdom; 9 Wolfson Palliative Care Research Centre, University of Hull, Hull, United Kingdom; 10 Hope Institute Hospital, Kingston, Jamaica; Alabama Psychiatry, UNITED STATES OF AMERICA

## Abstract

**Context:**

LGBTQIA+ people worldwide experience discrimination, violence, and stigma that lead to poor health outcomes. Policy plays a crucial role in ensuring health equity and safety for LGBTQIA+ communities. Given Lancet Commissions’ substantial impact on health policy across domains, we aimed to determine how LGBTQIA+ communities and their care needs are incorporated throughout Lancet Commission reports and recommendations.

**Methods:**

Using critical discourse analysis, we analyzed 102 Commissions for inclusion of and reference to LGBTQIA+ communities using 36 key terms. Three levels of analysis were conducted: 1) micro-level (overview of terminology use); 2) meso-level (visibility and placement of LGBTQIA+ references); and 3) macro-level (outlining characterizations and framing of references with consideration of broader social discourses).

**Findings:**

36 of 102 (35%) Commissions referenced LGBTQIA+ communities with 801 mentions in total. There were minimal (9/36) references made in the “Executive Summary,” “Recommendations,” and/or “Key Messages” sections of reports. LGBTQIA+ communities were most frequently discussed in reports related to HIV/AIDS and sexual and reproductive health. Few Commissions related to public health, or chronic conditions (9/60) referenced LGBTQIA+ communities. Some reports made non-specific or unexplained references; many discussed the LGBTQIA+ population without specific reference to sub-groups. LGBTQIA+ communities were often listed alongside other marginalized groups without rationale or a description of shared needs or experiences. We identified framings (legal, vulnerability, risk) and characterizations (as victims, as blameworthy, as a problem) of LGBTQIA+ communities that contribute to problematizing discourse.

**Conclusions:**

LGBTQIA+ people were rarely included in the Commissions, resulting in an inadvertent marginalization of their health needs. Policy initiatives must consider LGBTQIA+ groups from a strengths-based rather than problematizing perspective, integrating evidence-based approaches alongside community-based stakeholder engagement to mitigate inequities and promote inclusive care and policymaking.

## Introduction

**Fig 1:**
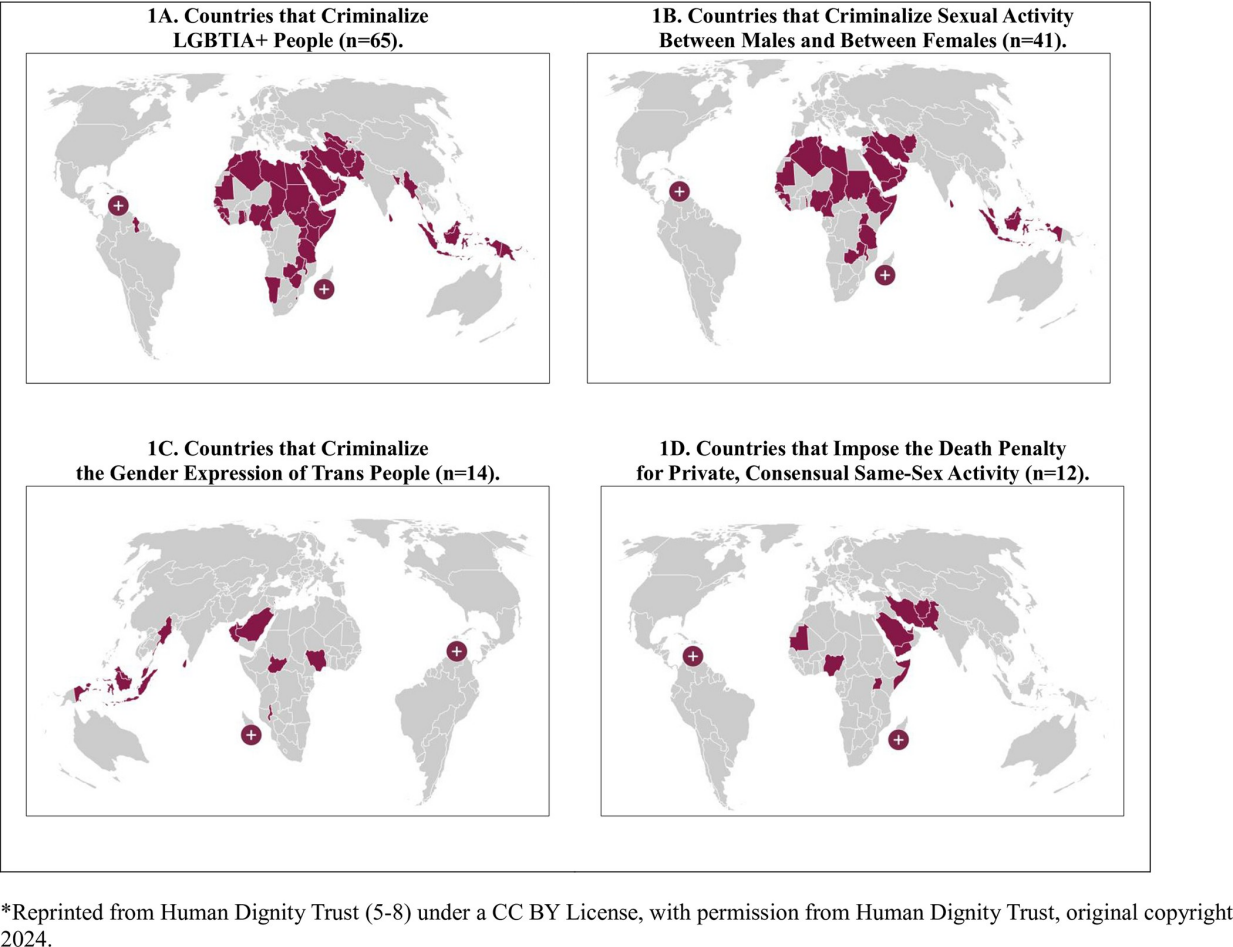
Maps of countries that criminalize LGBTQIA+ people.

A recent global survey revealed that 8% of adults have a minoritized sexual orientation, and 1% identify their gender as trans or nonbinary [1]. LGBTQIA+ people (lesbian, gay, bisexual, trans, queer/questioning, intersex, and asexual people, and anyone else who considers themselves to have a minoritized sexual orientation or gender identity) globally experience poorer health outcomes than the heterosexual and cisgender populations due to discrimination, stigma, violence, discriminatory policies, and other structural forces [2–4]. As of March 2024, globally 65 countries criminalize private, consensual, same-sex sexual activity, 12 countries have jurisdictions that can impose the death penalty for private, consensual same-sex activity, and 14 countries criminalize the gender identity and/or expression of trans people (Fig 1) [5–8]. Poorer physical, mental, and social health outcomes are just some of the far-reaching consequences of these longstanding structural inequities [2–4].

**Fig 2:**
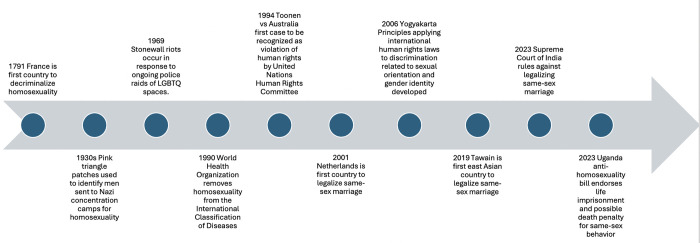
Cursory timeline summary of global LGBTQIA+ related policies, laws, and social activism events(9).

Health and social policy history is complex, has implications for health outcomes and social status, and disproportionately disadvantages LGBTQIA+ communities. Fig 2 provides a brief timeline of key policy and social activism milestones, advances and setbacks from a global perspective [9]. LGBTQIA+ people across the world face numerous health and healthcare inequities [10], including those that are shared by other marginalized groups and some that are unique to the LGBTQIA+ communities. Even in countries where LGBTQIA+ people are relatively visible, and policies prohibit anti-LGBTQIA+ discrimination, LGBTQIA+ individuals are still at increased risk of certain physical and mental health conditions. The experiences of discrimination and stigma LGBTIQA+ individuals experience are linked to higher incidence of serious illness (e.g., cancer, respiratory and cardiovascular disease [11–13]), higher rates of alcohol, tobacco and recreational drug use, poorer health outcomes, and higher rates of mental illness, including depression and suicidality [14,15]. Experiences and anticipation of discrimination within health settings also lead to delays in accessing healthcare, resulting in individuals presenting with more advanced disease [16,17]. Further, epidemiological data on health disparities for LGBTQIA+ individuals likely underestimate true disease prevalence and burden because data on sexual orientation and gender identity are not routinely collected [2]. Accurate data collection is poor for reasons ranging from lack of infrastructure (e.g., obsolete intake forms) to legal or safety concerns regarding collection, disclosure and documentation of these identities [18].

While LGBTQIA+ communities across the world experience marginalization, it is critical to emphasize that some are at greater risk than others. Specifically, LGBTQIA+ people with lower income [19]; who are children, youth [20], or older adults [21]; who are trans or nonbinary [22]; or from minoritized racial or ethnic groups [11,23] are all at greater risk for disparities and inequities in health and social care outcomes compared to LGBTQIA+ people writ large. Further, LGBTQIA+ individuals in countries that criminalize gender and sexual orientation diversity [5–8] not only experience the threat of day-to-day violence or legal action but also are at risk of developing health conditions due to being turned away from care [24,25] or avoiding institution-based healthcare out of mistrust and fear [26].

Critical risk factors for health and healthcare inequities for LGBTQIA+ communities are the criminalization of sexual and gender diversity, anti-LGBTQIA+ legislation, and lack of legal protections for LGBTQIA+ individuals in multiple countries. Such issues contribute to minority stress, stigma, inadequate or lack of access to care, and overt abuse or neglect [17], but also to a lack of research and knowledge on health and social care issues within LGBTQIA+ communities [27].

Numerous human rights organizations, including the United Nations [28] and the World Health Organization [29] have stated that the criminalization of and discrimination against those with minoritized gender identities or sexual orientations is a violation of basic human rights.

Despite these efforts, LGBTQIA+ communities continue to face vast inequities worldwide. Most recently, in 2024, Uganda’s Constitutional Court upheld laws and increased the severity of punishment for homosexual behavior, which in some cases can even include significant prison sentences and the death penalty [30,31]. Several neighboring countries are poised to follow suit [32]. Sweeping increases in anti-trans and anti-gay legislation across the United States led the Human Rights Campaign to declare a national state of emergency for LGBTQIA+ people in June 2023 [33]. Further, as of April 8^th^, 2024, the American Civil Liberties Union (ACLU) documented almost 500 anti-LGBT bills introduced in the US in 2024, with 101 related to healthcare. The very fact that laws exist that criminalize and discriminate against those of minoritized sexual orientation and gender identity contributes to a social climate which (re)produces significant stressors that LGBTQIA+ individuals must contend with daily.

### Rationale for current study

Increased advocacy and substantial, tangible improvements in healthcare for LGBTQIA+ communities are urgently needed worldwide. To achieve measurable improvements in health and social care outcomes for LGBTQIA+ communities, it is essential to understand whether and how research and policy documents describe these communities and their health needs. Increasingly, methods of documentary analysis are being utilized to analyze and appraise policy documents. A small but growing body of evidence has considered LGBTQIA+ communities within health policies, however often they are focused on specific conditions (e.g. cancer) [34], populations (e.g. aging LGBTQIA+ people) [35] or geographical regions [36]. In addition, many of these approaches draw on predominantly summative methods, and lack consideration of context or broader discourses. To date, analyses have also failed to consider policies with a global reach.

*The Lancet* and the broader family of Lancet journals (e.g., *The Lancet Child & Adolescent Health*, *The Lancet Public Health*, *The Lancet Global Health*, *The Lancet HIV*) maintain tremendous global influence. They are thus poised to advocate for minoritized groups. Lancet [37] journals have more than 36.8 million visits and 98.8 million downloaded articles each year via TheLancet.com and ScienceDirect, over 2.4 million social media followers, and over 275,000 annual news article mentions; Lancet podcasts have over 85,000 monthly listens, and audiences in 170 countries have viewed Lancet Webinars more than 5000 times.

*The Lancet* Commissions represent some of the highest-impact health policy products this family of journals produces. Through these multidisciplinary, multi-sector, international initiatives, academic experts partner with *Lancet* editors “*to identify the most pressing issues in science*, *medicine*, *and global health*, *with the aim of providing recommendations that change health policy or improve practice*.” Lancet Commission reports guide policymakers at international, national, and regional levels, making it critical they represent and advocate for the needs and experiences of marginalized and minoritized groups.

This article examines the extent to which Lancet Commissions have included LGBTQIA+ communities to date and how their needs and experiences have been represented in the reports. The overarching goal was to use these insights to develop recommendations for meaningful, respectful, and inclusive communication with, for, and about minoritized groups in the context of health policy. Specifically, using critical discourse analysis [38], we aimed to 1) examine the presence and context of references to LGBTQIA+ communities within published Lancet Commission reports and 2) consider how these references contribute to discourses that construct or shape characterizations, values, norms, or assumptions that may lead to the inclusion, exclusion, marginalization, and/or discrimination of LGBTQIA+ people [19].

## Methods

### Epistemological framework

We conducted a critical discourse analysis (CDA) of published Lancet Commission reports (hereafter called ‘Commissions’) to explore where and how they refer to LGBTQIA+ communities. Positioned within a social constructivist epistemological framework, CDA provides a means to systematically examine language and how it constructs and communicates power. It is, therefore, particularly useful for addressing issues of social justice and inequity [39]. In this context, discourse refers to spoken word or written text and is considered constitutive and a social practice [40]; lexical choices shape society and culture, and, reciprocally, society and culture shape language. Discourses also have the power to shape worldviews and thus to construct, legitimize, and perpetuate social inequities, as well as reproduce and transform society and culture [38]. As an example, every time a homophobic slur is used, and goes unchallenged, it contributes to reproducing and sustaining homophobia in society and culture.

### Analysis

#### Phase one: Identification of commissions, keyword search and data extraction

The Commission reports were identified from the Lancet website, which hosts the entire archive (www.thelancet.com/commissions). All commissions published in Lancet journals from conception through November 25^th^, 2023 (*n* = 102) were extracted [41]. Four authors (MB, SWG, MM, WER) identified and coded key terms in all published reports. The coding team identified 32 key terms by consulting the literature and discussing the relevance of each term to discourse around LGBTQIA+ social justice issues. Key search terms can be found in Table 1. Two coders (MB, WER) then individually searched the text of each Commission for the terms. The coders created an entry in an Excel database for every mention of a key term in a Commission. Each entry included the page number of the Commission where the key term appeared and a reproduction of the paragraph where the key term was mentioned. Coders also indicated whether the key terms appeared in the “Key Messages,” “Executive Summary,” or “Recommendations” sections of the Commission. The total number of references to each key term in each Commission was tallied. A third coder (MM) conducted a confirmatory check of a randomly selected 10% of excluded Commissions (with no key term mentions) to ensure no references to LGBTQIA+ communities had been missed.

**Table 1:** Search terms and descriptive statistics.

Search Key Term	Frequency of Term	Percentage of Total Number of Mentions (%)	Total Number of Commissions that Mention Term	Number of Mentions in Each Commission	Average Mentions per Commission (of Those that Mention)
MSM	153	19.10	5	30, 76, 34, 12, 1	30.6
Sexualit*	101	12.61	15	48, 6, 9, 1, 3, 14, 4, 4, 2, 2, 2, 2, 2, 1, 1	6.7
Gender identit*	53	6.62	9	19, 1, 17, 5, 2, 3, 1, 2, 3	5.9
Sexual and gender identit*	14	1.75	1	14	
Gay	55	6.87	14	3, 30, 4, 5, 3, 1, 1, 1, 2, 1, 1, 1, 1, 1,	3.9
Sexual orientat*	55	6.87	18	22, 2, 4, 8, 2, 1, 1, 2, 2, 1, 2, 1, 1, 1, 2, 1, 1, 1,	3.1
Sexual and gender minorit*	45	5.62	4	41, 1, 2, 1	11.3
Sexual minorit*	36	4.49	9	1, 6, 1, 1, 8, 12, 5, 1, 1	4.0
Transgender	36	4.49	13	4, 6, 1, 12, 1, 1, 3, 1, 1, 1, 2, 1, 2	2.8
Men who have sex with men	36	4.49	10	3, 3, 1, 7, 7, 3, 6, 3, 2, 1	3.6
Bisexual	24	3.00	10	1, 13, 1, 1, 1, 1, 3, 1, 1, 1	2.4
SOGIE	23	2.87	2	6, 17	11.5
Same(-)sex	22	2.75	7	6, 2, 5, 3, 3, 2, 1	3.1
Lesbian	19	2.37	11	2, 3, 2, 1, 1, 1, 4, 2, 1, 1, 1	1.7
LGBTI	19	2.37	2	10, 9	9.5
Gender minorit*	14	1.75	2	9, 5	7.0
Key population*	23	2.87	8	1, 2, 3, 4, 3, 2, 7, 1	2.9
Homosexual*	12	1.50	8	1, 4, 2, 1, 1, 1, 1, 1	1.5
LGBT	10	1.25	2	7, 3	5.0
LGBTQI	9	1.12	1	9	9.0
Homophobi*	7	0.87	5	2, 1, 1, 1, 2	1.4
Intersex	6	0.75	5	1, 2, 1, 1, 1	1.2
Sex characteristics	6	0.75	4	3, 1, 1, 1	1.5
Transphobia	5	0.62	4	1, 1, 1, 2	1.3
LGBTQ	5	0.62	2	1, 4	2.5
Queer	2	0.25	2	1, 1	1.0
Gender and sexual minorit*	2	0.25	2	1, 1	1.0
Non-binary	2	0.25	2	1, 1	1.0
LGBTQIA+	1	0.12	1	1	1.0
Transgendered	1	0.12	1	1	1.0
Transsexual	1	0.12	1	1	1.0
Gender orientat*	1	0.12	1	1	1.0
Questioning	1	0.12	1	1	1.0
Sexual and gender orientat*	1	0.12	1	1	1.0
Same(-)gender	1	0.12	1	1	1.0
Asexual	0	0.00	0	0	0.0
**Total**	**801**	**100.00**	* *		

#### Phase two: Critical discourse analysis

The Commission reports were analyzed using the Fairclough approach to CDA [38], which draws on discourse analysis techniques to examine lexical items within a dataset across different levels. This commenced with a textual (micro) level analysis, considering the item itself and the words collocated around or proximally located to it. In the context of this analysis, the items analyzed at the micro level were each usage of the ‘key terms’ within every Commission. The analysis then progressed to the contextual (meso) level, exploring the visibility and placement of the references within the Commission and how LGBTQIA+ communities were addressed alongside other groups. Finally, the analysis moved to a societal (macro) level to consider the broader implications of the reference for the genre (a form of discourse with socially agreed upon conventions^5^—in this context, peer-reviewed scientific journals) and society.

The analysis included consideration of intertextuality, which examines how and where the document references other sources of knowledge (in this case, citations), and interdiscursivity, which explores the discourses drawn upon in the document. Intertextuality was considered through a systematic and iterative process of ‘zooming in and out’ and moving backward and forward through the dataset (i.e., each Commission and the collection as a whole) to examine explicit and latent constructions of LGBTQIA+ inclusion or exclusion. We also considered notable absences where references to the terms were expected but not found, such as in reports examining sexual health.

#### Application

The analysis (conducted August-November 2023) was led by KB (a sociolinguist), who trained a subset of authors (the CDA team: DB, NM, SWG) in the methods. Analysis was conducted in three phases: 1) exploratory phase, 2) description and interpretation and 3) explanatory phase.

*Exploratory phase*. The analysis methods were trialed using three sample Commissions, which were analyzed independently by each member of the CDA team. The analysis involved: (1) consideration of which specific lexical terms were used to reference LGBTQIA+ communities (e.g., ‘LGBT,’ ‘gay,’ ‘men who have sex with men,’ ‘transgender,’ etc.); (2) examination of how each reference might contribute to broader discourses that construct or shape characterizations, values, norms, or assumptions that may lead to the inclusion, exclusion, marginalization, or discrimination of LGBTQIA+ people (interdiscursivity); and (3) consideration of the citations provided as evidence for statements made regarding LGBTQIA+ communities (intertextuality). After completing this initial analysis, the team met to discuss their findings. A second phase of exploratory analysis was conducted using three different Commissions with varying subject matters and number of references to LGBTQIA+ communities. The team reconvened to discuss the analysis, refine analytical processes, and ensure consistency among the team members. At this stage, the team began considering potential broader themes, interpretations, and implications emerging from the initial findings.

*Description and interpretation*. Following the initial exploratory phase, the team developed an extraction table in Excel to collate example quotations across emergent themes. The entire corpus of 36 Commissions, including those used in the exploratory phase, was divided between the four members of the CDA team and analyzed independently at the micro and meso levels, as described above. The CDA team met two more times to consider the macro analytic level and identify and synthesize key findings and interpretations. During these meetings, four additional key terms were identified (‘nonbinary,’ ‘same-sex,’ ‘same-gender,’ and ‘sexuality,’ the latter pertaining to sexual orientation, not intimacy), which had not been included in the initial extraction phase, bringing the total number of key terms to n = 36. All Commissions were then re-searched by the phase one analysis team for the use of these terms to ensure completeness (see Table 2 for the complete list of key terms).

**Table 2:** Mentions and descriptive statistics by commission report.

Commission Report	Commission Topic of Focus	Year of Commission	Total Number of Search Key Term Mentions in Commission (Total N = 801)	Number of Different Search Key Terms Mentioned	Search Key Terms Mentioned (Number of Mentions of Term)	Key Term Mentioned in Key Messages or Executive Summary
Accelerate progress—sexual and reproductive health and rights for all: report of the Guttmacher–Lancet Commission	Sexual and reproductive health and rights	2018	126	16	LGB* (LGBTQI, 9); Lesbian (2); Gay (3); Bisexual (1); Transgender (4); Queer (1); Intersex (1); Homophobi* (2); Sexual minorit* (1); Sexual orientation (22); Gender identit* (19); Sex characteristics (3); Men who have sex with men (3); Key population* (1); Same(-)sex (6); Sexualit* (48)	Yes (Sexual orientation, Gender identities, Sexuality)
Advancing global health and strengthening the HIV response in the era of the Sustainable Development Goals: the International AIDS Society—Lancet Commission	HIV/AIDS	2018	118	16	LGB* (LGBTI, 10); Lesbian (3); Gay (30); Bisexual (13); Transgender (6); Intersex (2); Men who have sex with men (3); MSM (30); Key population* (2); Sexual orientation (2); Gender identit* (1); Sexual minorit* (6); Homophobi* (1); Transphobia (1); Same(-)sex (2); Sexualit* (6)	No
The Lancet commission on peaceful societies through health equity and gender equality	Health equity; gender equity	2023	106	13	Transgender (1); Men who have sex with men (1); SOGIE (6); Sexual orientation (4); Sexual and gender minorit* (41); Gender identit* (17); Sexual and gender identit* (14); Homophobi* (1); Transphobia (1); Gender minorit* (9); Sex characteristics (1); Gender and sexual minorit* (1); Sexualit* (9)	Yes (Sexual and gender minorities)
Sexually transmitted infections: challenges ahead	Sexual and reproductive health and rights; sexually transmitted infections	2017	92	6	MSM (76); Gay (4); Bisexual (1); Homosexual* (1); Men who have sex with men (7); Key population* (3)	Yes (Men who have sex with men)
A UNAIDS–Lancet Commission on Defeating AIDS—Advancing Global Health	HIV/AIDS	2015	73	9	MSM (34); Gay (5); Transgender (12); Transgendered (1); Homosexual* (4); Men who have sex with men (7); Key population* (4); Same(-)sex (5); Sexualit* (1)	Yes (MSM, Men who have sex with men, Transgender)
Women, power, and cancer	Women’s health; cancer	2023	45	13	Sexual orientation (8); Gender identit* (5); Bisexual (1); Sexual minorit* (1); Sexual and gender minorit* (1); Lesbian (2); Gay (3); LGB* (LGBTQ, 1); Transgender (1); Homophobi* (1); Transphobia (1); SOGIE (17); Sexualit* (3)	Yes (Sexual orientation, Gender identity)
Our future: a Lancet commission on adolescent health and wellbeing	Child/adolescent health	2016	33	10	LGB* (LGBT, 7); Lesbian (1); Gay (1); Bisexual (1); Transgender (1); Homosexual* (2); Sexual minorit* (1); Sexual orientation (2); Same(-)sex (3); Sexualit* (14)	Yes (LGBT)
The Lancet Psychiatry Commission on intimate partner violence and mental health: advancing mental health services, research, and policy	Mental health; untimate partner violence	2022	29	14	Lesbian (1); Gay (1); Transgender (3); Non-binary (1); Homosexual* (1); Homophobi* (2); Sexual and gender minorit* (2); Gender and sexual minorit* (1); Gender minorit* (5); Sexual orientation (1); Gender identit* (2); Transphobia (2); Same(-)sex (3); Sexualit* (4)	Yes (Gender and sexual minorities)
The UCL–Lancet Commission on Migration and Health: the health of a world on the move	Migration	2018	24	9	LGB* (LGBTI, 9); Lesbian (1); Gay (1); Bisexual (1); Transsexual (1); Intersex (1); Sexual minorit* (8); Sex characteristics (1); Same(-)gender (1)	No
Women and Health: the key for sustainable development	Women’s health	2015	27	7	Lesbian (4); Gay (2); Bisexual (3); Homosexual* (1); Sexual minorit* (12); Sexual orientation (1); Sexualit* (4)	No
A future for the world’s children? A WHO-UNICEF-Lancet Commission	Child health	2020	20	10	LGB* (LGBT, 3); Queer (1); Questioning (1); Intersex (1); Transgender (1); Non-binary (1); Sexual minorit* (5); Sexual orientation (2); Gender identit* (3); Sexualit* (2)	Yes (Gender identities)
Accelerating the elimination of viral hepatitis	Liver disease; viral hepatitis	2019	19	4	MSM (12); Homosexual* (1); Men who have sex with men (3); Key population* (3)	No
Lancet Commission on the Value of Death	Death and dying	2022	11	7	LGB* (LGBTQIA+, 1); Lesbian (2); Gay (1); Bisexual (1); Sexual orientation (2); Same(-)sex (2); Sexualit* (2)	No
The legal determinants of health: harnessing the power of law for global health and sustainable development	Health policy	2019	10	10	Lesbian (1); Gay (1); Bisexual (1), Transgender (1); Intersex (1); Sexual minorit* (1); Sexual orientation (1); Gender identit* (1); Sex characteristics (1); Same(-)sex (1)	No
The EASL-Lancet Commission: Protecting the next generation of Europeans against liver disease complications and premature mortality	Liver disease	2021	9	3	MSM (1); Men who have sex with men (6); Key population* (2)	No
Essential Medicines	Access to medicines	2017	7	1	Key population* (7)	No
The Lancet Commission on the future of care and clinical research in autism	Autism	2021	7	4	Transgender (1); Sexual orientation (2); Gender identit* (2); Sexualit* (2)	No
Public policy and health in the Trump era	Health policy	2021	6	3	LGB* (LGBTQ, 4); Gay (1); Sexual orientation (1)	No
The Lancet and Financial Times Commission on governing health futures 2030: growing up in a digital world	Health policy; technology	2021	6	3	Sexual orientation (1); Gender identit* (3); Sexualit* (2)	No
The Future of Psychiatry	Mental health	2017	5	4	Lesbian (1); Gay (1); Transgender (2); Sexual or gender orientation (1)	No
The Lancet Commission on global mental health and sustainable development	Mental health	2018	5	5	Lesbian (1); Gay (1); Bisexual (1); Transgender (1); Sexual orientation (1)	No
Liver diseases in the Asia-Pacific region: a Lancet Gastroenterology & Hepatology Commission	Liver disease	2020	3	1	Men who have sex with men (3)	No
The Lancet commission on medicine, nazism, and the Holocaust	History of Medicine; Nazism and the Holocaust	2023	3	2	Sexual and gender minorit* (1); Sexual orientation (2)	No
Addressing liver disease in the UK: a blueprint for attaining excellence in health care and reducing premature mortality from lifestyle issues of excess consumption of alcohol, obesity, and viral hepatitis	Liver disease	2014	2	1	Men who have sex with men (2)	No
Ending stigma and discrimination in mental health	Mental health	2022	2	1	Sexualit* (2)	No
The Lancet women and cardiovascular disease Commission: reducing the global burden by 2030	Cardiovascular disease; women’s health	2021	2	1	Transgender (2)	No
A Lancet Commission on 70 years of women’s reproductive, maternal, newborn, child, and adolescent health in China	Sexual and reproductive health and rights; child health	2021	2	2	Homosexual* (1) Key populations (1)	No
Culture and health	Culture’s impact on health	2014	1	1	Gender orientation (1)	Yes
Global Cancer Surgery: pragmatic solutions to improve cancer surgery outcomes worldwide	Cancer	2023	1	1	Sexual orientation (1)	No
High-quality health systems in the Sustainable Development Goals era: time for a revolution	Health systems strengthening	2018	1	1	Sexual orientation (1)	No
Institutionalisation and deinstitutionalisation of children	Child health	2020	1	1	Sexual orientation (1)	No
The expanding role of primary care in cancer control	Cancer; primary care	2015	1	1	Sexualit* (1)	No
The Lancet Commission on diagnostics: transforming access to diagnostics	Diagnostics; technology	2021	1	1	Sexual minorit* (1)	No
The path to healthy ageing in China: a Peking University–Lancet Commission	Health in China; ageing	2022	1	1	Sexualit* (1)	No
The path to longer and healthier lives for all Africans by 2030: the Lancet Commission on the future of health in sub-Saharan Africa	Health in Africa	2017	1	1	Homosexual* (1)	No
The Tsinghua-Lancet Commission on Healthy Cities in China: unlocking the power of cities for a healthy China	Health in China	2018	1	1	Men who have sex with men (1)	No

*Explanatory phase*. The CDA team developed a shared working document to construct an initial narrative of the findings, supported by examples from the data, and to expand upon the potential implications of this work for the genre and at a societal (macro) level. The authors’ intention was to identify examples of best practices and delineate ways to improve communication with, for, and about minoritized groups at a policy level. As such, a list of recommendations for practice and policy derived from the data was also developed at this stage. Finally, the CDA team met with the entire study team (all authors) to present the findings and discuss the description and interpretation, after which the analysis was refined and finalized.

### Rigor, integrity, and ethical considerations

Throughout the data extraction and analysis, processes were embedded to ensure rigor and integrity [42]. All Commission reports were subject to a systematic search to identify key terms and a random selection of 10% of commissions were recoded to ensure no items had been missed. The critical discourse analysis was conducted by a diverse range of researchers and clinical academics (sociolinguist, sociologist, nurse scientist, medical student), with expertise in qualitative and quantitative methods. We purposefully trialed, discussed, and refined the analytical process to enable reflexivity, minimize potential biases, and ensure rigor. Our detailed methodological description ensures transparency and replicability of the processes undertaken, and the inclusion of examples from the Commissions evidences the credibility and reliability of the findings. Since the data analyzed were publicly available policy documents, the risks in terms of harm and confidentiality were minimal. However, due to the sensitive nature of this research, and the focus on minoritized groups, caution was taken to ensure LGBTQIA+ people and commissioners were described in thoughtful and respectful terms to minimize potential harm and ensure an accurate representation of discourses.

## Results

One-hundred and two (N = 102) Commissions were analyzed (Table 2). Overall, 35% (36/102) of Commissions referred to LGBTQIA+ communities. Of these, 25% (9/36) included references in the “Executive Summary,” “Recommendations,” and/or “Key Messages” sections. The number of references in each Commission report that included them ranged from 1–126.

### Findings from the critical discourse analysis

#### Micro-level findings

*Terminology*. The lexicon used to refer to LGBTQIA+ communities varied across Commissions (Table 1). Terminology included commonly used descriptors of sexual orientation (e.g., ‘lesbian,’ ‘gay,’ ‘bisexual,’ ‘asexual’), as well as more outdated terms (e.g., ‘homosexual’). Additionally, common descriptors of gender identity (e.g., ‘transgender,’ ‘intersex’) and rare instances of outdated and stigmatizing terms (e.g., ‘transsexual,’ ‘transgendered’) were identified, along with a variety of abbreviations (e.g., ‘LGBT,’ ‘LGBTQ,’ ‘LGBTI,’ ‘SOGIESC’). Further descriptors used to refer to LGBTQIA+ communities were associated with behaviors (e.g., ‘men who have sex with men,’ ‘MSM,’ ‘same-sex’ relationships, etc.).

Descriptors were also employed to position LGBTQIA+ individuals in terms of risk (e.g., ‘key populations,’ ‘high-risk populations,’ ‘at-risk populations’) and to differentiate them from other (dominant groups of) people, as evident in the use of terms like ‘sexual minorities’ or ‘gender minorities.’ Some examples used language that was othering and implied an assumed norm (e.g., ‘sexual or gender non-conforming,’ ‘gender-atypical,’ and ‘not heterosexual’). The most extreme examples used stigmatizing and archaic terminology (e.g., ‘sexual deviancy’).

Some more uncommon descriptors were also identified incidentally during the analysis which were used to discuss LGBTQIA+ women, such as ‘female partnerships’ and ‘female couples’. These desexualized descriptors were particularly noteworthy when used within a Commission focusing on sexual and reproductive health and rights and contrast with the most common term found across Commissions: ‘men who have sex with men (MSM).’ Moreover, there was occasional inconsistency between and conflation of gender identity and sexual orientation, as seen, for example, in a reference to ‘gender orientation’.

In instances where abbreviations were employed it was rare for Commissions to recognize the sub-identities and varying risk profiles within and beyond the abbreviation. There were also examples where an abbreviation was used interchangeably with another non-synonymous term (e.g., ‘LGBTI’ and ‘sexual minorities’). Finally, on rare occasions, abbreviations were presented in a less common format (e.g., ‘LGBQT,’ ‘SOGIESC’) without an explanation of the choice.

The terminology used throughout the Commissions varied in frequency and lexical choice. However, the decisions behind these choices were rarely made explicit. For example, authors tended not to state their positionality in relation to the lexicon used or the socio-political context behind their choice of language despite accepted guidelines from professional organizations (e.g., the American Psychological Association’s ‘Inclusive Language Guide’) [43]. An explicit and exemplary approach was evident in the Commission on ‘Women, Power, and Cancer’ [44], which delineated sex and gender and clarified the approach adopted (i.e., intersectional feminist).

#### Meso-level findings

*Visibility*. References to LGBTQIA+ communities were most prevalent in Commissions related to HIV/AIDS and sexual and reproductive health and rights (>60 references per Commission report). They were also frequently identified in those related to childhood and adolescence, intimate partner violence, migration, and women’s health (18–43 references per report). Few, if any, references to LGBTQIA+ people were included in Commissions related to public health agendas, chronic non-malignant conditions, and cancer care (except for two Commissions on women and cancer, which included 27 [45] and 45 [44] references, respectively) regardless of the increased prevalence of these conditions for LGBTQIA+ people. For example, despite the growing evidence to suggest an increased prevalence of cardiovascular disease among LGBTQIA+ populations [46,47], the Commission on ‘Women and Cardiovascular Disease’ did not mention LGBTQIA+ people [48]. In contrast, other minoritized groups, including along axes such as ethnicity and socioeconomic status, were discussed in detail in the Commission, further bringing into relief the exclusion of LGBTQIA+ people. Table 3 provides the top five commissions ranked by the number of key term mentions.

**Table 3:** Ranking of top five commissions by number of key term mentions.

Rank	Title of Commission	Topic of Commission	Total Number of Key Term Mentions
**1**	Accelerate progress—sexual and reproductive health and rights for all: reporting of the Guttmacher-Lancet Commission	Sexual and Reproductive Health and Rights	126
**2**	Advancing global health and strengthening the HIV response in the era of the Sustainable Development Goals: The International AIDS Society-Lancet Commission	HIV/AIDS	118
**3**	The Lancet commission on peaceful societies through health equity and gender equality	Health and Gender Equity	106
**4**	Sexually transmitted infections: challenges ahead	Sexual and Reproductive Health and Rights	95
**5**	A UNAIDS-Lancet Commission on Defeating AIDS—Advancing Global Health	HIV/AIDS	73

Within the Commissions that did mention LGBTQIA+ people, not all sub-communities were discussed, and some garnered much more focus than others. For example, when an LGBTQIA+ community was discussed in greater depth, the focus was often on ‘men who have sex with men (MSM)’ in the context of infectious diseases, especially sexually transmitted infections. The bisexual community was rarely considered independently but more often mentioned alongside the lesbian or gay communities. In some cases, examples of bisexual erasure were evident; this was notable in the context of Commissions on women’s health [44], with comparisons of statistics between lesbian and heterosexual people making no mention of bisexual women, who are at especially high risk of experiencing outcomes common to women, such as intimate partner violence [49–51].

It is important here to distinguish visibility (as a proxy for inclusion) and meaningful engagement with the needs of LGBTQIA+ people. Many Commissions (65%) did not refer to LGBTQIA+ people within the report, or they displayed an active decision to exclude certain groups—for example, excluding trans women from a report ‘owing to the current paucity and quality of data’ despite evidence to the contrary [48]. Many additional Commissions were unclear in their positionality, for example, failing to clarify whether they included trans individuals in discussions of ‘men’ and ‘women.’

However, other Commissions made vague references to LGBTQIA+ communities, for example, mentioning sexual orientation only in passing and without discussion of its relevance. In one notable example, found in a Commission that had one of the highest numbers of total references (n = 106) to LGBTQIA+ people and which claimed a focus on gender equality [52], sexual orientation and gender identity were conflated throughout the report. There were descriptions of “women and sexual and gender minorities.” The Commission also failed to discuss the needs of LGBTQIA+ people, apart from in one location where a panel was used to highlight a specific geographical example.

*Placement.* Concerning the placement of references to LGBTQIA+ communities, they were seldom mentioned in the “Executive Summaries” of the Commissions. Where LGBTQIA+ people were mentioned in the “Executive Summary,” it was either as part of Commissions focused on sexual or reproductive health and rights or within a list of other minoritized groups without an explanation as to why they had been grouped together.

It was also rare for LGBTQIA+ people to be mentioned in the “Key Messages” or “Recommendations” sections of Commissions (25%). This was often the case even when the needs of LGBTQIA+ communities had been addressed in reasonable depth elsewhere in the Commission (e.g., in the context of viral hepatitis or HIV/AIDS Commissions). Outside of the context of Commissions concentrating on HIV/AIDS, references to LGBTQIA+ communities tended to be limited to sections on inequities, discrimination, or sexual and reproductive health and rights. In several instances, all mentions of LGBTQIA+ communities in a Commission were within a single sentence, paragraph, or panel.

*Grouping*. LGBTQIA+ communities were often included as part of lists with other minoritized or marginalized groups or communities. While such grouping could yield a positive impact in terms of visibility, it also risks contributing to the collectivization of minoritized and marginalized groups, ignoring the unique needs of each and the intersectional needs of those belonging to multiple such groups and identities.

#### Macro-level findings

*Framing*. **Legal frame.** One example was the placement of LGBTQIA+ communities within a legal frame (see Table 4, Quotation 1). In these cases, sexual orientation was mentioned alongside, for example, ethnicity, disability, religion, family or marital status, or other legally protected characteristics (Quotation 2), such as those protected by the UK Equality Act, 2010 [53,54].

**Table 4:** Macro-level framings of LGBTQIA+ communities with exemplar quotations from Lancet commission reports.

Quotation number	Type of grouping or characterisation	Quotation
**GROUPINGS**		
1	Legal Frame	“Governments often exclude a wide swath of vulnerable people from high quality services, including asylum seekers, refugees, undocumented immigrants, expatriate workers, indigenous peoples, nomadic people, or groups that are historically marginalised because of sexual orientation, gender identity, sex characteristics, disability, political beliefs, or religious affiliation.” Commission #92 (2019)
2	Legal Frame	“These abuses (including harassment, cyberbullying by peers, threats of sexual violence, and body shaming), which are often motivated by race and ethnicity, sexual orientation, or gender identity, are estimated to have increased during the COVID-19 pandemic.” ‘Commission #69 (2019)
3	Vulnerability Frame	*“Violations of children’s rights are common across many domains*, *such as poverty; inadequate nutrition; violence and war; gender bias and discrimination against sexual minorities; poor access to clean water*, *shelter*, *education*, *and health services; and climate degradation and unsustainable use of planetary resources*.*”* Commission #3 (2020)
4	Vulnerability Frame	"Furthermore, vulnerable or marginalised groups, such as MSM and PWID, risk criminal prosecution given that homosexuality is illegal in several countries in the region." Commission #7 (2019)
5	Vulnerability Frame	“High rates of IPV [intimate partner violence] are also experienced by other groups, including sexual and gender minorities, people with disabilities, migrants, and people from marginalised ethnic or Indigenous groups.” Commission #88 (2022)
6	Vulnerability Frame	"People with different cultural and religious backgrounds or sexual or gender orientation have differing needs and perceived obstacles to approaching conventional mental health-care services. Inadequate cultural sensitivity within a health-care environment (e.g., no prayer rooms for patients of different faiths) and insufficient cultural competencies in mental health professionals (e.g., services that do not address specific needs of lesbian, gay, and transgender communities) can result in reduced acceptability and accessibility of services for these populations." Commission #77 (2019)
7	Risk Frame	“Scaling up testing to achieve the diagnosis rates required for elimination may be possible without widespread population testing. Targeted screening approaches need to focus on high-risk groups, including PWID, individuals who are incarcerated, and MSM, with universal screening offered in relevant settings such as prisons, supervised injecting centres, homeless or migrant centres, or opioid substitution centres.” Commission #7 (2019)
8	Risk Frame	*“People with*, *or at risk of developing*, *liver disease frequently belong to highly stigmatised groups*. *These include individuals with obesity*, *people with alcohol use disorders*, *PWID [people who inject drugs]*, *people who are incarcerated*, *immigrants*, *and* **men who have sex with men.”** **Commission #8 (2021)**
9	Risk Frame with Recognition of Structural Causes	“The many reasons why certain groups are more vulnerable to HIV infection vary widely between countries and between communities; the reasons are rarely linear or singular. High-risk sexual behaviour might play a part, but the reasons often stem from stigma, human rights violations, gender inequality, violence against women, criminalisation, inappropriate legislation and policies, and poor leadership and political courage, all of which prevent access to HIV services. In some environments, poverty and restricted livelihood options drive the epidemic; elsewhere, HIV transmission is higher in wealthier segments of society. Each country needs a detailed analysis of its at-risk populations and hot spots.” Commission #5 (2015)
**CHARACTERISATIONS**		
10	Victim	*“Men and adolescent boys are more likely to be recruited*, *often deceptively*, *for various forms of strenuous manual labour*, *including commercial fishing and construction*.*74* **Sexual minorities** *who are trafficked are often subjected to forced sex work*.*”* Commission #97 (2018)
11	Victim	“Violence against, and victimisation of, sexual minorities begin at an early age. Childhood sexual and physical abuse are more frequently experienced by sexual minority women than by heterosexual women, which might be due to gender-atypical behaviour, substance misuse, and running away from home.” Commission #102 (2015)
12	Victim	“We cannot ignore the risks that the internet poses for women and gender minorities, as harassment and misogyny are amplified online.” Commission #71 (2023)
13	Blameworthy	“Transgender women are more likely to acquire HIV than most adults of reproductive age, and 19% of transgender women are estimated to be living with HIV (the effect of HIV on transgender men has yet to be established). Transgender women who sell sex and inject drugs are at an even greater risk of acquiring HIV.” Commission #5 (2015)
14	Blameworthy	“Sexual transmission of HCV is less efficient than that of HBV. Incidence is very high among some HIV infected MSM and associated with both high risk sexual and recreational drug use practices.” Commission #7 (2019)
15	Blameworthy	*“Treatment optimism about the benefits of improved cART [combination antiretroviral therapy] has been associated with increased risky behaviour; MSM with stronger perceptions that cART has reduced the threat from HIV infection and reduces the need for safer sex engage more often in risky behaviours*, *such as non-condom receptive anal intercourse*.*”* Commission #61 (2017)
16	Blameworthy	*“People identifying as LGBQT+ have increased rates of preventable deaths and face barriers accessing health services*.*”* Commission #42 (2022)
17	Problem	*“Anti-retroviral treatment (ART) fundamentally changed the course of the epidemic by substantially reducing mortality from HIV infection and as part of HIV control strategies*. *However*, *many populations around the world are still highly affected by HIV*, *particularly young women in southern and eastern Africa*, *men who have sex with men (MSM)*, *sex workers*, *and injecting drug users*. *Additionally*, *there are concerning signs of complacency and setbacks in countries and populations that had previously made substantial progress*. *[*.* *.* *.*] People individually have an important role*. *Whenever possible*, *individuals need to take responsibility for prevention*, *know their HIV status*, *and minimise the risk of infecting others*. *Because HIV is intertwined with sex and complicated drivers of behaviour*, *it will always be difficult to control*. *This explains why*, *for example*, *gay men in high-income countries with very low levels of discrimination*, *no or few legal obstacles*, *and free access to services have much higher infection rates than the general population*. *Biological (transmission through anal sex) and behavioural factors (more sexual partners) drive the high infection rates in this group*, *along with social marginalisation (official government underfunding of the AIDS response among gay people in many rich countries) in even the most open societies*.*”* Commission #5 (2015)
18	Problem	*“Disparate health risks and outcomes are a shared experience of sexual minority women*, *although the extent of health disparity seems to vary by age*, *ancestry*, *culture*, *society*, *economics*, *and nationality*, *and no epidemiological data exist for the health of sexual minority women worldwide*.*”* Commission #102 (2015)

#### Vulnerability frame

Another common grouping concerned perceived ‘vulnerability,’ ‘marginalization,’ or ‘disadvantage’ (Quotation 3). In these instances, LGBTQIA+ people were listed, for example, alongside migrants, people with low socioeconomic status, victims of violence and war, and others vulnerable to deprivation of their human rights or criminalization (Quotation 4). However, it was scarce to find any explanation as to why these communities had been grouped (Quotation 5). Commissions largely omitted any indicators of shared experiences among these groups and did not provide an in-depth explanation of why they might have similar specific needs. The analysis was generally limited to acknowledging that these groups may have unmet needs (Quotation 6).

#### Risk frame

A further frequent grouping was about ‘risk’ and suggested ‘high-risk’ or ‘risky’ behaviors (Quotation 7). Here, LGBTQIA+ people were repeatedly listed alongside people who inject drugs, sex workers, and people in juvenile detention centers or prisons, especially in the context of being at risk of contracting or transmitting infectious diseases, particularly HIV/AIDS and/or viral hepatitis. Again, it was uncommon for there to be any explanation as to why these groups were listed together, beyond all being conceived of as ‘high-risk’ or ‘key populations’ for the transmission of certain infectious diseases (Quotation 8). Nevertheless, a few Commissions provided more nuanced analyses for the reasons behind ‘concentrated epidemics’ or ‘hot spots’ and potentially higher infectious disease transmission rates in communities such as MSM, including by considering the roles of structural factors (Quotation 9). Commissions that did address structural factors as drivers of infectious disease transmission were primarily those focused on HIV/AIDS.

*Characterization*. How LGBTQIA+ communities were referenced and grouped with other communities played a role in how they were characterized.

#### As victims

It was common for LGBTQIA+ people to be characterized as victims, for example, as being the targets of ‘discrimination,’ ‘abuse,’ ‘stigma,’ and/or ‘marginalization’ (Quotation 10). However, there was seldom mention of any structural, social, or individual agents in relation to these phenomena, such as homophobia, transphobia, etc. Such phenomena were most often mentioned in Commissions on HIV/AIDS, where the structural determinants of discrimination and stigma were occasionally addressed more comprehensively.

Another example of the victim frame concerned violence, with a few Commissions linking being LGBTQIA+ with attracting violence. While higher levels of violence must be discussed and prioritized within Commissions, on occasions Commissions directed the blame for these actions toward the individuals themselves based on their personal circumstances and characteristics. Indeed, it was rare for the potential structural causes of violence against LGBTQIA+ people, to be indicated by Commissions (Quotation 11).

There were also notable absences related to violence and discrimination toward LGBTQIA+ people. One Commission specifically addressing gender-based discrimination and violence failed to mention or acknowledge violence directed toward gender expansive people, such as trans and nonbinary people, despite widespread reports of increasing levels of violence experienced by these groups [55–57]. Elsewhere in the Commission, despite referring to ‘women *and gender minorities*,’ and recognizing misogyny as a structural cause of violence, there was a failure to mention transphobia as a cause (Quotation 12).

#### As blameworthy

Another common framing was that of blame (Quotation 13). This was often, though not exclusively, linked to sexual behaviors and underscored by a subtext of moral judgment (Quotation 14). For example, LGBTQIA+ people were referred to as facing inequities ‘*because of*’ their ‘non-conforming’ sexual orientation or gender identity. This may appear to imply that inequities are an inherent and unavoidable consequence of being LGBTQIA+ rather than rooted in societal or cultural patterns of discrimination, evident in phraseology such as ‘sexual orientation is a *source* of discrimination’ [45].

In cases related to infectious diseases, such as HIV/AIDS, LGBTQIA+ people were frequently described as voluntarily engaging in risk behaviors and, by implication, as worthy of blame. They were portrayed as transmitters of infection, as choosing not to use condoms (Quotation 15), and as causing increased rates of ‘preventable deaths’ (Quotation 16). The blame-framed presentation tended to occur when Commissions had a broader health agenda but included a subsection on sexual and reproductive health and rights. Notably, in Commissions specifically focused on HIV/AIDS or sexually transmitted infections, this presentation was less common, with increased recognition of the structural causes contributing to observations of increased prevalence.

#### As a problem

Linked to the blame frame was a problematizing one employed more subtly throughout the Commissions. This framing tended to position LGBTQIA+ people as a challenge or problematic in some way, with their health concerns described as being a ‘problem’ or ‘issue.’ Communities were also sometimes characterized as lacking responsibility or being complacent, for example, in the context of increasing rates of HIV infection. (Quotation 17).

At a micro level, the use of terms that assume a societal norm contribute to this framing (e.g., ‘non-conforming gender identities or sexual orientations,’ ‘when not heterosexual,’ ‘if they do not conform to social norms regarding sexual orientation and gender identity,’ etc.). On rare occasions, Commissions also employed a discourse of biological imperative, which reinforces this problematizing frame with respect to LGBTQIA+ communities. Notably, a Commission on ‘Women and Health’ included the statement, ‘Nulliparity and reduced contraceptive pill use increase their [sexual minority women’s] risk of breast cancer and uterine cancer’ [45], problematizing and presupposing LGBTQIA+ women’s bodily choices and experiences.

At a meso level, the problematizing frame was also attended to through broad statements about a lack of evidence concerning LGBTQIA+ communities. This contributed to the perception that clear avenues for improving policies and practices for LGBTQIA+ populations may be difficult or impossible to achieve. Commissions variously described the ‘paucity and quality of data’ [48], a lack of ‘data disaggregated by age and sex or data on high-risk populations’ [58], or how ‘some stratifications might be sensitive to collect in certain contexts, such as for race, ethnicity, religion, or sexual orientation’ [59]. Some Commissions recognized the contribution of stigma and discrimination to LGBTQIA+ people’s experiences. However, many Commissions’ focused on ‘under-reporting’ and individuals’ purported lack of willingness to disclose LGBTQIA+ identities—without any presentation of potential solutions. This shifts the locus of responsibility more toward LGBTQIA+ people to disclose their identities and provide these data, despite the risks that may entail, or risk inhibiting their access to care.

This problematizing frame and the frequent shifting of responsibility toward LGBTQIA+ people were also utilized in ways that undermined or minimized the needs and experiences of LGBTQIA+ individuals. For example, in the context of HIV/AIDS, one Commission discussed an increased incidence of HIV in gay men ‘despite an increasingly open and tolerant attitude toward homosexuality’ [58], contributing to both the problematizing and blame narratives, wherein LGBTQIA+ communities themselves are designated as the source of the problem and as worthy of blame. This problematizing frame was extended with reference to the financial burden to society of ‘key populations’ (e.g., ‘If the political will to spend so much public money on injecting drug users, MSM, and sex workers exists, the bulk of these countries should have the fiscal capacity to pay for their national HIV programs’) [58].

In addition, such minimizing and undermining were also evident in other contexts where the validity of LGBTQIA+ experiences was questioned. For example, one Commission discussed the *‘perceived* obstacles’ [60] that LGBTQIA+ people may experience in accessing mental health services, and another appeared to use the paucity of evidence argument to undermine the differences in risks and outcomes that LGBTQIA+ people experience and their causes (Quotation 18).

Finally, many Commissions exhibited limited utilization of citations to substantiate statements related to LGBTQIA+ communities when they were considered.

## Discussion

Our analysis demonstrates that Lancet Commission reports often underrepresented the needs of LGBTQIA+ communities. When these communities were addressed, it was primarily concerning individuals’ sexual and reproductive health, with most emphasis on HIV/AIDS. Sex and reproductive-related health outcomes were suggested to stem from sexual orientation and gender identity rather than the structural and systemic forces that lead to inequities. There was minimal reference to LGBTQIA+ communities in the context of public health issues such as cancer and respiratory, cardiovascular, and liver disease, despite evidence of an increased prevalence of these conditions amongst LGBTQIA+ people. We argue that limiting conceptions of LGBTQIA+ individuals to these singular aspects of their identity provides, at best, an incomplete picture of their experiences and needs. At its worst, such reductionism causes harm by failing to recognize how programs, policies, and interventions targeting other aspects of health and well-being could be tailored to LGBTQIA+ communities.

Additionally, explicit mentions of member groups within LGBTQIA+ communities often characterized them principally or exclusively in terms of their perceived association with behaviors that expose them to risks for certain health statuses or outcomes. These were almost universally framed using a deficit discourse [61]. The behaviors in question were frequently related to sexual or substance use activity, and the outcomes were often infectious diseases. It is important to acknowledge the disproportionate rates of many such outcomes experienced by LGBTQIA+ communities [62]. However, the omission of nearly every Commission on the structural factors that shape these outcomes, as well as the strengths inherent in LGBTQIA+ communities, obscure crucial opportunities for intervention and policy creation to support and improve LGBTQIA+ health.

### Limitations

There are strengths and limitations to this work. To our knowledge, this is the first in-depth analysis of Commission reports to investigate the inclusion and multidimensional contextualization of a particular population (i.e., LGBTQIA+ people) with implications for policy and research. The identification, extraction, and analysis of Commission reports were systematic and rigorous, and in the decision to use critical discourse analysis, the study went beyond beyond a summative presentation toward meaningful engagement with the impact of these reports on broader societal discourses. However, due to the age of some of the Commissions, and the advances in equity, diversity, belonging, inclusion, and justice initiatives, references made to LGBTQIA+ communities in the Commissions reports may not be reflective of the attitudes of the commissioners today. In addition, while the *Lancet* journals and *Lancet* Commissions are prestigious and have a global reach, it is beyond the scope of this work to examine the extent to which these reports impact local, regional, national, and/or international health policies and health care delivery.

### Implications and future research

The language we employ in scientific and policy writing is fundamentally shaped by societal and cultural conceptions, beliefs, and biases; reciprocally, language shapes society and culture [38,63]. Discourses have the power to shape the way people view the world. The *Lancet* Commissions are a global force for positive change, with a track record of improving millions of people’s lives and health and social care worldwide through rigorous data analysis, international expert collaboration, and innovative recommendations for policy and practice. Yet the language used in many of the Commissions, though selected by experts in the field of health, may contribute to LGBTQIA+ communities being negatively viewed by the healthcare community, policymakers, and societies at large. Moreover, the claim of a purported ‘lack of evidence’ pertinent to LGBTQIA+ communities is not sufficient justification for continued omission from any scientific or policy document. Instead, insisting on the inclusion of under-researched groups experiencing minoritization and marginalization may spotlight high-quality evidence where it exists while bringing much-needed attention to areas where there is limited robust evidence for public health action in these communities. Further, the superficial inclusion of minoritized communities in these Commissions, without meaningful engagement with their needs and suitable responses to them, may be as damaging as exclusion and erasure.

Alongside authors of a recent *Lancet HIV* article on ‘Putting people first in communication about HIV’ [64], we advocate for person-first language that “puts people before their condition” (p. e623) and, we would add, before any single aspect of their identity. We also advocate a shift from stigmatizing or blaming language, which contributes to a deficit model, toward less value-laden and more assets- or strengths-based terminology. Recognizing that language is constantly evolving, we believe that Commissions should take a proactive approach to ensuring the use of current terminology while remaining receptive to future changes [65]. Involving community members or experts from minoritized or marginalized groups in the Lancet Commission teams is one way to support this approach in the future. Additionally, increased transparency about the decision-making processes for author inclusion and those authors’ positionalities may help readers interpret report implications and recommendations.

Beyond the context of Commission initiatives, additional research should consider employing critical discourse analysis to evaluate other policy documents for the inclusion of and reference to other historically marginalized, minoritized, and at-risk groups (e.g., persons with disabilities; persons experiencing homelessness; incarcerated persons; persons with stigmatized or systemically excluded racial, cultural, ethnic, and religious identities). Such analyses should also be conducted at local and national levels to increase policy transparency, identify contextually relevant health and social care inequities, close policy to practice gaps in care delivery settings, and hold policy makers accountable for current legislation and regulations that perpetuate systemic, institutional, and interpersonal discrimination. More broadly, as investigative reams adopt research approaches that employ a social justice lens to answer scientific questions (e.g., community-based participatory research) [66–69], this will also increasingly facilitate the development of equitable, sustainable, and mutually beneficial partnerships with communities [70,71]. In the long-term, such approaches and partnerships will ultimately lead to community-centered knowledge generation and research conceptualization, design, and conduct to inform more inclusive, evidence-based policies. In short, inclusive policies capable of promoting health equity must be based on inclusive evidence rooted in a social justice approach [72,73].

## Conclusions

In summary, we encourage critical thinking and discussion regarding how our findings and recommendations may be transferable to other communities that experience minoritization and marginalization (Table 5). Despite their different experiences and needs, other minoritized and marginalized communities may be subject to similar or different omissions and problematization in Commission reports as LGBTQIA+ communities. Therefore, we advocate for thoughtful examination of how we can, ‘center the margins’ and bring all communities with lived experiences of minoritization and marginalization, especially along intersecting axes, to the forefront of Commissions [74,75]. It is critical to recognize that minoritized and marginalized positions in society are not, and never have been, inherent; they are socially constructed [76,77]. Although we cannot erase the injustices faced by minoritized and marginalized communities, discourses that risk reproducing and compounding harms can be minimized and changed. We, as members of society, alongside Lancet commissioners as experts with power and influence in health fields, have an opportunity to effect that change. We should not fail to use it.

**Table 5:** Recommendations for best practices for addressing LGBTQIA+ communities and other groups experiencing minoritisation and marginalisation in the Lancet commissions.

1. Make deliberate and specific language choices; say who is being referred to by using the precise term (e.g., gay and bisexual men; transgender women) rather than using the ‘LGBTQIA+’ umbrella term.
2. Strive to be inclusive of all LGBTQIA+ groups to avoid contributing to the erasure of some LGBTQIA+ populations.
3. Ensure that gender-specific Commissions adequately include transgender and non-binary communities and other gender expansive individuals as well as cisgender people.
4. Where terms are used that group different LGBTQIA+ communities in some way (e.g., sexual minorities, men who have sex with men), be explicit as to why these terms are being used and consult members of these communities before settling on nomenclature.
5. Strive to avoid tokenistic or superficial inclusion, such as including minoritised or marginalised groups in a list but failing to discuss their needs (and suitable responses to them) any further.
6. If grouping different minoritised or marginalised populations is necessary, provide clarification for the rationale, especially when combining communities for whom the similarities are not self-evident (e.g., ‘racial and ethnic minorities’ and ‘sexual minorities’).
7. Avoid language that implies blame or moral judgment towards minoritised or marginalised individuals or groups.
8. When employing a victim framing, acknowledge the structural forces that may contribute to the victimisation of minoritised or marginalised individuals in society.
9. Refrain from problematising minoritised or marginalised individuals and groups, unless accompanied by an explanation of the issue’s origins, including structural factors, and potential solutions.
10. Recognise the importance of intersectionality and that, for example, being LGBTQIA+ is only one facet of peoples’ lives and health.
11. Avoid perpetuating associations between minoritised or marginalised communities and issues related to those aspects of their identities exclusively (e.g., being LGBTQIA+ and sexual behaviours), instead recognising their holistic health and social care needs.
12. Comprehensively address LGBTQIA+ groups across a broader spectrum of topics, encompassing public health agendas on subjects such as non-communicable diseases, psychological health and well-being, and others.
13. Substantiate claims about LGBTQIA+ populations with sufficient and robust evidence and citations.
14. Where inadequate evidence precludes the inclusion of content related to minoritised or marginalised groups, including LGBTQIA+ communities, be specific about future research needs and directions rather than simply stating that there are insufficient data.
15. In Commissions with multiple authors, consider the ‘voice’ and ensure consistency in how LGBTQIA+ communities are referred to and framed throughout the Commission.
16. Adopt a transparent and inclusive approach to Commissioner selection to increase equity in opportunities for involvement and ensure minoritised and marginalised groups are referred to with appropriate language, dignity, and respect.
